# Regulation of Cdc42 for polarized growth in budding yeast

**DOI:** 10.15698/mic2020.07.722

**Published:** 2020-05-19

**Authors:** Kristi E. Miller, Pil Jung Kang, Hay-Oak Park

**Affiliations:** 1 Department of Molecular Genetics, The Ohio State University, Columbus, OH 43210.; 2 Present address: Department of Biochemistry and Cell Biology, The Geisel School of Medicine at Dartmouth, Hanover, NH 03755.

**Keywords:** cell polarity, Rho GTPase, Saccharomyces cerevisiae, spatial cue-directed cell polarization, positive and negative feedback regulation, cell cycle

## Abstract

The Rho GTPase Cdc42 is a central regulator of cell polarity in diverse cell types. The activity of Cdc42 is dynamically controlled in time and space to enable distinct polarization events, which generally occur along a single axis in response to spatial cues. Our understanding of the mechanisms underlying Cdc42 polarization has benefited largely from studies of the budding yeast *Saccharomyces cerevisiae*, a genetically tractable model organism. In budding yeast, Cdc42 activation occurs in two temporal steps in the G1 phase of the cell cycle to establish a proper growth site. Here, we review findings in budding yeast that reveal an intricate crosstalk among polarity proteins for biphasic Cdc42 regulation. The first step of Cdc42 activation may determine the axis of cell polarity, while the second step ensures robust Cdc42 polarization for growth. Biphasic Cdc42 polarization is likely to ensure the proper timing of events including the assembly and recognition of spatial landmarks and stepwise assembly of a new ring of septins, cytoskeletal GTP-binding proteins, at the incipient bud site. Biphasic activation of GTPases has also been observed in mammalian cells, suggesting that biphasic activation could be a general mechanism for signal-responsive cell polarization. Cdc42 activity is necessary for polarity establishment during normal cell division and development, but its activity has also been implicated in the promotion of aging. We also discuss negative polarity signaling and emerging concepts of Cdc42 signaling in cellular aging.

## INTRODUCTION

**Figure 1:**
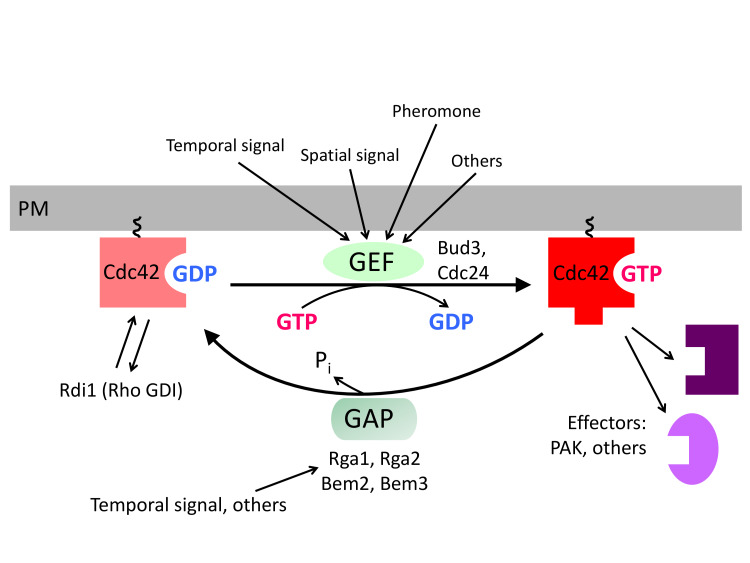
FIGURE 1: The Cdc42 GTPase and its regulators in budding yeast. Cdc42 activity is regulated by at least three types of regulators: the GEFs, Cdc24 and Bud3; the GAPs, Rga1, Rga2, Bem2, and Bem3; and the GDI Rdi1. These regulators are likely to mediate the regulation of Cdc42 in response to internal and external signals. Cdc42 regulates the organization of the actin and septin cytoskeletons and polarized secretion via its downstream effectors including PAKs.

Cells within a multicellular organism or unicellular organisms such as yeast and bacteria exhibit some form of polarity in order to carry out specialized functions. Cell polarization involves polarized organization of cell shape or cellular components including organelles, proteins, or RNAs. Establishing and maintaining polarity is essential for cellular processes including differentiation, chemotaxis, morphogenesis, cell movement, and cell division [1, 2]. The highly conserved Rho GTPase Cdc42 regulates polarity development across the eukaryotic kingdom. Like other Rho GTPases, Cdc42 cycles between an active GTP-bound state and an inactive GDP-bound state. Guanine-nucleotide exchange factors (GEFs) activate Cdc42 by catalyzing the exchange of GDP for GTP, whereas GTPase activating proteins (GAPs) enhance its intrinsic GTPase activity, facilitating the conversion of Cdc42 to its inactive form. The active Cdc42 recruits and/or regulates a variety of downstream effectors (**Figure 1**; **Table 1**). Localized activation of Cdc42 has been shown to be a key event leading to cell polarization in yeast and mammalian cells [3, 4]. While the universal role of Cdc42 in establishing and maintaining tissue/cell polarity is critical for normal cell division and is also implicated in tumor suppression, Cdc42 activation has been suggested to contribute to tumor cell invasion and migration as well as cellular aging [5–7]. Thus, uncovering the molecular mechanisms underlying Cdc42 polarization will provide insights into how cell growth is regulated and how perturbation of Cdc42 activity may lead to disease and aging.

**Figure 2:**
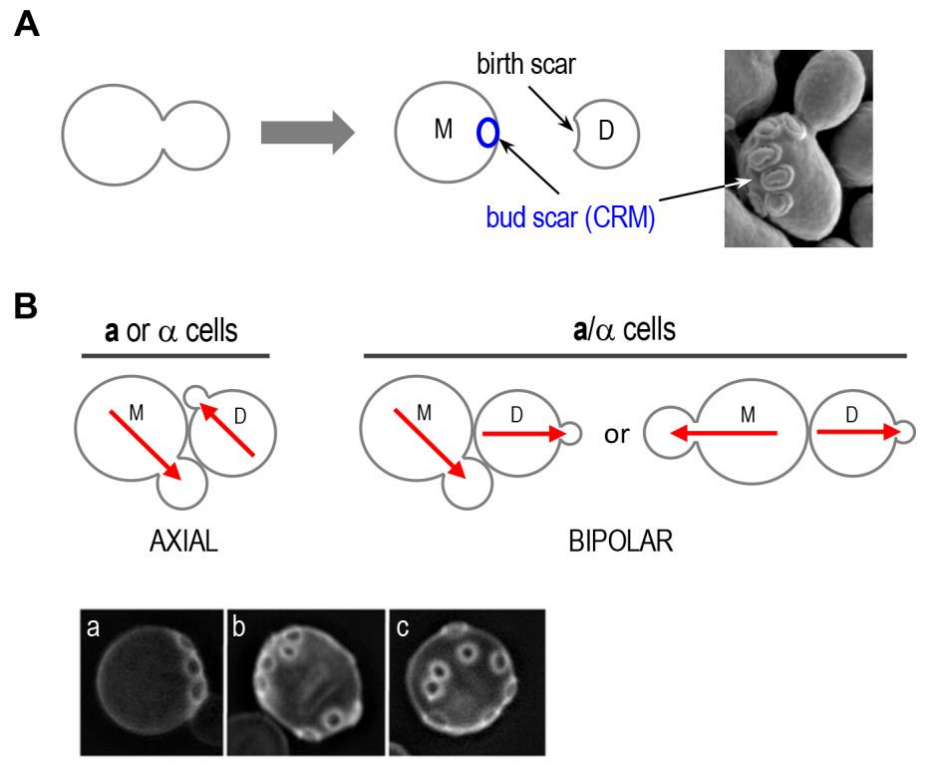
FIGURE 2: Oriented cell divisions of *S. cerevisiae*. **(A)** Each cell division leaves a bud scar that marks the site of division on the mother cell surface and birth scar on daughter cell. Successive divisions produce distinct patterns of bud scars (also called cytokinesis remnants: CRMs). Bud scars can be visualized by scanning electron microscopy (image on the right) or by staining with the dye Calcofluor (as shown in **B**). Electron micrograph was taken from Ref. [44] with permission. **(B)** Axial and bipolar patterns of bud-site selection in *S. cerevisiae*. Red arrows denote the axes of cell polarization. Below, the patterns of bud scars on the yeast cell surface resulting from the different modes of budding are visualized after Calcofluor staining: (a) axial pattern; (b) bipolar pattern; and (c) random budding by a mutant (such as *rsr1*Δ). Micrographs were published previously [20].

Polarized growth is generally directed by intracellular or extracellular spatial cues such as cell-cell contacts, chemoattractants, and cortical landmarks. Budding yeast is an excellent model system to study cell polarity because it displays pronounced cell polarization during various phases of its life cycle, such as budding during vegetative growth and mating between two haploid cells of opposite mating types. During the mitotic cell cycle, yeast cells undergo oriented cell division by choosing a specific bud site depending on their cell type. Haploid **a** and α cells bud in an axial pattern, in which both mother and daughter cells select a new bud site adjacent to their immediately preceding division sites. In contrast, diploid **a**/α cells bud in the bipolar pattern, in which daughter cells predominantly bud at the pole distal to the division site, and mother cells can choose a new bud site near either pole [8–10] (**Figure 2**). These different patterns of growth require cell-type-specific cortical cues and the Rsr1 GTPase module, composed of Rsr1 (also known as Bud1), its GAP Bud2, and its GEF Bud5 [11–15]. Multiple genetic and physical interactions suggest that the Rsr1 GTPase module guides Cdc42 and its regulators to direct organization of the actin cytoskeleton and septin filaments for polarized growth to the selected site [11, 13, 16–21].

**TABLE 1.** The Cdc42 and Rsr1 GTPses and their regulators and effectors.

**Name of protein or protein complex**	**Protein activity**	**Function in relation to Cdc42 polarization in haploid cells**	**Selected References**
Cdc42	Rho GTPase	Essential for polarity establishment and maintenance	[61, 87, 106–109]
Cdc24	GEF for Cdc42	Essential for polarity establishment and maintenance Activates Cdc42 likely after Start	[17, 19, 39, 41, 42, 54, 63, 72, 109–115]
Bud3	GEF for Cdc42; Axial landmark	Essential for axial budding of haploid **a** and α cells Activates Cdc42 before Start	[14, 37, 40, 116, 117]
Rga1	GAP for Cdc42	Essential for preventing polarization within old cell division sites	[44, 46, 49, 58, 59, 94]
Rga2, Bem2, Bem3	GAP for Cdc42 (and Rho1)	Important for polarity regulation and septin ring assembly	[60, 88, 89, 94, 118–123]
Rdi1	GDI for Cdc42 (and Rho1 and Rho4)	Important for cycling of Cdc42 and other Rho GTPases between the cytosol and PM during cell polarization	[75–77, 124, 125]
Bem1	Scaffold for GEF-based amplification of Cdc42 activation	Forms a complex with Cdc24 and PAK (Cla4/Ste20) important for robust Cdc42 polarization after Start; Interacts with Exo70 and Rsr1-GDP	[17, 21, 63, 64, 68, 71, 72, 109, 118, 126, 127]
Cla4	PAK (p21-activated kinase) Effector of Cdc42	Part of Bem1 polarity complex important for Cdc42 polarization	[63, 70, 72, 113, 128–130]
Ste20	PAK, Effector of Cdc42	Part of Bem1 polarity complex important for Cdc42 polarization	[65, 70, 131–133]
Gic1, Gic2	Related effectors of Cdc42	Act in parallel with Rsr1 in Cdc42 polarization prior to Start Stabilize Cdc42-GTP on the PM	[61, 87, 134–137]
Rax1, Rax2	Interdependent transmembrane proteins	Localize to the bud tip and division site; Stably inherited at old division sites; Anchor negative polarity cues Nba1 and Nis1; Important for proper targeting of the bipolar landmark.	[34, 49, 100, 101]
Aim44 (= Gps1)	Bud neck associated protein	Negative regulator of Cdc42 signaling at the division site that functions in parallel with Rga1; Scaffold for Nba1 and Nis1	[98, 138]
Nba1, Nis1	Negative polarity complex	Anchored by Rax1/2 at old cell division sites Recruits Rga1 via direct interaction between Nba1 and Rga1	[34, 49]
Bni1	Formin, nucleates actin filaments; Effector of Cdc42 and Rho1; Polarisome component	Promotes the assembly of actin cables and actin rings, important for exocytosis, cytokinesis, and spindle orientation.	[139–147]
Exo70	Exocyst subunit	Required for the tethering of post-Golgi vesicles to the PM Interacts with Cdc42, Bem1, and Rho3	[64, 148–150]
Bud4, Axl1, and Axl2 (including Bud3)	Axial landmark	Intrinsic positional marker for Cdc42 polarization in haploid **a** and α cells	[14, 84–86, 117, 151–157]
Rsr1 (=Bud1)	Ras GTPase	Essential for proper bud-site selection Interacts with Cdc24, Cdc42, and Bem1 Promotes Cdc42 polarization by linking the spatial cue to Cdc42 prior to Start	[11, 14, 17, 18, 20, 21, 61, 67]
Bud2	GAP for Rsr1	Essential for proper bud-site selection	[12, 15, 46, 158–160]
Bud5	GEF for Rsr1	Essential for proper bud-site selection Interacts with the axial and bipolar landmark	[13, 86, 161–163]

Polarized growth and cytokinesis in budding yeast are coordinated with cell cycle progression. Yet how polarity establishment is temporally regulated in the G1 phase is still largely unknown. The cyclin-dependent kinase Cdc28 (CDK1), complexed with a G1 cyclin, is required to promote bud emergence as well as other key events in the cell cycle (such as DNA replication and spindle pole body duplication) once cells pass through the irreversible commitment point known as ‘Start' [22, 23], which is equivalent to ‘restriction point' in mammalian cells. The Start transition corresponds to the time of the nuclear exit of approximately 50% of Whi5, a transcriptional repressor [24], which partitions the G1 phase into two temporal steps. The first step (T_1_) is critical for cell size control and depends on the upstream cyclin Cln3 [25]. Whi5, which is functionally analogous to mammalian retinoblastoma (RB), inhibits two heterodimeric transcription factor complexes SBF (Swi4–Swi6) and MBF (Mbp1–Swi6), which dictate G1/S transcription. Phosphorylation of Whi5 initially by Cln3-CDK1 drives its exit from the nucleus, leading to activation of the G1/S transcriptional program, including expression of the G1 cyclins Cln1 and Cln2, which trigger further inactivation of Whi5 [26, 27]. The second step (T_2_), defined from Start to bud emergence, is cell size-independent [25]. Recent studies have uncovered an intricate crosstalk among the polarity proteins that function in these distinct steps in the G1 phase to promote Cdc42 polarization at the proper time and place, which will be a major focus of this review (see below).

In the absence of spatial cues or Rsr1, yeast cells can still direct polarized growth to a single random site. This spontaneous cell polarization without spatial cues (often referred to as ‘symmetry breaking') is thought to rely on positive feedback loops that promote the amplification of small stochastic clusters of Cdc42. One of these positive feedback mechanisms may rely on actin-based transport of Cdc42, whereas the other involves the Cdc42-signaling network that includes a complex of the scaffold protein Bem1, Cdc24 (a Cdc42 GEF), and a Cdc42 effector p21-activated kinase (PAK), although the details of these mechanisms are still under debate. *In vivo* analyses and computational modeling have also suggested a negative feedback loop that enhances robustness to the polarity circuit. Symmetry breaking has been covered in greater detail in a number of reviews [28–31], and we refer interested readers to these sources and references therein. These key players in symmetry breaking are discussed later in this review with respect to their role in spatial-cue-dependent polarization.

Another key aspect of yeast budding is asymmetric cell division, resulting in mother and daughter cells with distinct characteristics including mother cell-specific aging [32]. Mother cells progressively age and produce a finite number of daughter cells, referred to as replicative lifespan (RLS). In contrast, daughter cells are born with full replicative potential. Interestingly, however, aged cells sometimes undergo symmetric cell division, and thus daughter cells from very old mothers often display reduced lifespans [33]. Negative polarity factors in Cdc42 signaling have been implicated in yeast aging [34]. Yet the causal factors or consequence of aging still remain elusive [35]. In this review, we discuss polarity establishment during yeast budding. In particular, we focus on recent findings that cover regulation of Cdc42 in relation to the two temporal phases of G1. We also discuss the importance of negative polarity signaling and the possible implication of Cdc42 signaling in cellular aging.

## BIPHASIC CDC42 POLARIZATION IN THE G1 PHASE

### The first step determines the axis of cell polarity

**Figure 3:**
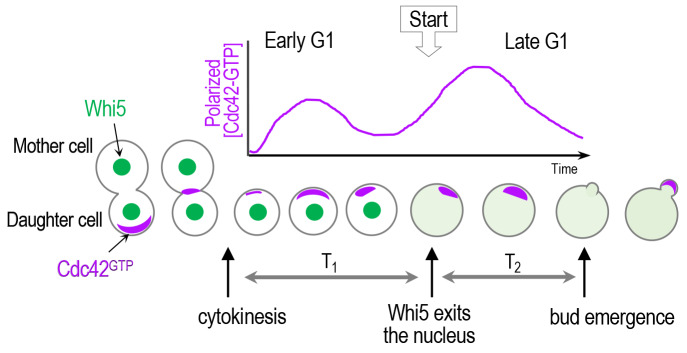
FIGURE 3: A scheme of biphasic Cdc42 polarization in the G1 phase. Cdc42 polarization occurs stepwise triggered by its two GEFs: first by Bud3 and subsequently by Cdc24 [37]. Whi5 partitions the G1 phase into two temporal steps, and the ‘Start' transition corresponds to the time of the nuclear exit of approximately 50% of Whi5 [24]. The sites of Cdc42 polarization prior to the onset of cytokinesis and until a new bud appears are marked with purple color.

Haploid **a** and α cells select a new bud site adjacent to the previous division site. This axial budding pattern depends on the deposition of a transient cortical landmark, referred to as the axial landmark, composed of Bud3, Bud4, Axl1, and Axl2 (see [36] and references therein). While earlier studies suggested a morphogenetic hierarchy from spatial cues to Cdc42 polarization via the Rsr1 GTPase module, our unexpected finding of Bud3 as a Cdc42 GEF has uncovered a more complex regulatory mechanism underlying Cdc42 polarization in correlation with cell cycle progression [37]. Bud3 contains a conserved Dbl homology (DH) domain, which is necessary for GEF activity of Rho GEFs [38], and functions as a GEF for Cdc42 both *in vitro* and *in vivo* [37]. Prior to this finding, Cdc24 had been known as the sole Cdc42 GEF in budding yeast [39]. Bud3 localizes to the mother-bud neck (i.e., future cell division site), peaking in M phase, and stays at the division site until the next G1 phase [40]. In contrast, the majority of Cdc24 is sequestered in the nucleus in late M and early G1 phases via interaction with the nuclear anchor Far1 in haploid cells [41, 42]. Consistent with these localization patterns, Bud3 is mainly responsible for activation of Cdc42 in early G1, accounting for Cdc42 polarization soon after cytokinesis, while Cdc24 activates Cdc42 in late G1. The distribution and activity of Cdc42 *in vivo* has been quantitatively defined by live-cell imaging using a fluorescent probe carrying a PBD (p21-binding domain), which contains CRIB (Cdc42/Rac-interactive binding motif) and specifically interacts with Cdc42-GTP in budding yeast [43–45]. Using this biosensor, we showed that yeast cells with a mutation in the Bud3 DH domain with defective GEF activity display greatly diminished Cdc42 polarization in early G1 compared to wild type (WT). In contrast, a temperature sensitive *cdc24* mutant was able to polarize Cdc42 normally in early G1 but failed in subsequent Cdc42 polarization and arrested as unbudded cells at the non-permissive temperature [37]. Importantly, this study provided the first evidence for stepwise Cdc42 polarization in correlation with two temporal steps in the G1 phase (**Figure 3**).

As a component of the axial landmark complex, Bud3 likely functions in liking spatial information from the cell division site to the next bud site by triggering the initial activation of Cdc42 for polarity establishment in haploid cell types. Then, how is a single, new bud site established near the last division site even though Bud3 (and other components of the axial landmark) form a ring at the division site? This question is especially relevant because the perimeter of the ring appears large enough to accommodate multiple sites. A subsequent study from our group [46] addressed this question and uncovered the potential involvement of negative and positive feedback loops in selection of a proper bud site during axial budding. Live-cell imaging showed that the Cdc42-GTP level fluctuates around the septin ring until the axis of Cdc42 polarization becomes stabilized in mid G1. This wandering behavior of Cdc42-GTP cluster is particularly evident in daughter cells during the first phase of G1, which lasts much longer in daughter cells compared to mother cells. The output of delayed negative feedback, where accumulation of a molecule leads to its own dispersal so that the concentration does not reach a steady state, results in oscillatory or wandering dynamics of a molecule [43, 47, 48]. To explain these distinct dynamics of Cdc42-GTP cluster in mother versus daughter cells, we used mathematical modeling. We considered a generic model of particle density of the membrane bound Cdc42 on a two-dimensional computational domain with the axial landmark as a ring in the center. Initially, we assumed two phases to mimic two temporal steps of the G1 phase with two sequential positive feedback loops. We also considered the distribution of the Cdc42 GAP Rga1 that localizes as a ring at the division site to inhibit Cdc42 re-polarization. However, simulations from this initial biphasic modeling with the fixed distribution of Rga1 as a homogeneous ring at the division site led to budding within the division site. Surprisingly, by reexamining Rga1 localization, we found that Rga1 exhibits a fragmented ring-like structure or amorphous distribution at the division site during cytokinesis and G1 phase. This time-dependent Rga1 distribution at the division site is necessary for proper bud-site selection [46]. We thus implemented this new pattern of Rga1 localization and assumed putative delayed negative feedback together with positive feedback in the first phase in our biphasic model. Importantly, when we assumed transient negative feedback with different durations to mimic the lengths of the first temporal step of G1 in mother and daughter cells (typically about 3 min in mother cells and 15 min in daughter cells) in addition to sequential positive feedback loops, this modeling recapitulated the distinct Cdc42 polarization dynamics in mother versus daughter cells as observed *in vivo* [46]. Therefore, pre-Start Cdc42 polarization likely involves negative and positive feedback loops that link the axial landmark to Cdc42 polarization [37, 46]. Furthermore, the correlation of spatial distribution of Rga1 with cell cycle progression is important to fine-tuning the axis of cell polarity in budding yeast [46, 49].

Polarity proteins often exhibit oscillatory or wandering behavior, as observed in plants [50], mammalian cells [51], fission yeast [47], and budding yeast [46, 52]. Thus, delayed negative feedback may be a common mechanism underlying control of GTPase function in many organisms. In theory, there could be two possible scenarios of how active Cdc42 induces its own inactivation: Cdc42-GTP activates its inhibitor (e.g., a Cdc42 GAP) or inhibits its activator (e.g., a Cdc42 GEF). It has been suggested that negative feedback might involve inactivation of the Cdc24 GEF during symmetry breaking in diploid budding yeast [53]. However, this is unlikely to occur in haploid cells during the first phase of G1 because the majority of Cdc24 is sequestered in the nucleus until Start [41, 42, 54]. While a critical study has yet to uncover the precise mechanism, negative feedback during pre-Start Cdc42 polarization in haploid cells may potentially involve the Cdc42 GEF Bud3 or its GAP Rga1. Bud3 contains numerous putative phosphorylation and ubiquitination sites [55–57], but it is currently unknown how Bud3 is regulated to promote Cdc42 activation specifically prior to Start. Rga1 is also required for proper bud-site selection [58–60] and prevents re-budding at the same site by inactivating Cdc42 from the division site [44], although its regulation is not fully understood (see below).

**Figure 4:**
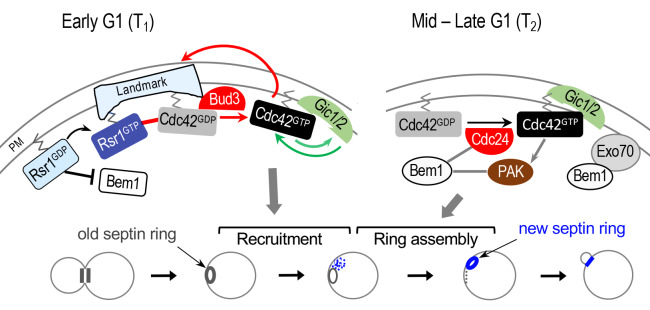
FIGURE 4: Model for biphasic Cdc42 polarization coupled to stepwise assembly of a new septin ring during axial budding. During T_1_, two positive feedback loops likely operate: one involving Bud3 and Rsr1 (marked with red arrows and line); another involving Gic1/2 (marked with green arrows). The Bem1-mediated positive feedback loop likely operates during T_2_ (gray lines and arrows). For simplicity, some other known links are omitted, including the link between the axial landmark and the Rsr1 GTPase module; and the link between Rsr1-GTP and Cdc24 (see text). Stepwise assembly of the septin ring in correlation with Cdc42 polarization during each temporal step in G1 is shown below: gray and blue rings denote old and new septin rings, respectively. Blue dots and gray dotted line denote newly recruited septin ‘clouds' and disassembling old ring, respectively. Modified from Refs. [61] and [21].

Does biphasic Cdc42 polarization occur only in cells budding in the axial pattern? Is stepwise activation of Cdc42 necessary to ensure sequential execution of the processes in the G1 phase? Our recent study that included analyses of temperature-sensitive *cdc42* alleles found that cells cannot traverse the G1 phase without Cdc42 polarization prior to Start [61]. Remarkably, a positive feedback loop involving two redundant Cdc42 effector proteins Gic1 and Gic2 is likely to act in parallel with Rsr1 in Cdc42 polarization prior to Start (**Figure 4**). As suggested in a previous report [62], cells lacking Rsr1 and both Gic proteins fail to polarize despite the presence of all components implicated in symmetry breaking. Specifically, depletion of Rsr1 and both Gic1 and Gic2 prevents cells from polarizing Cdc42 during the first phase of G1 and results in cell cycle arrest. Genetic data and FRAP (fluorescent recovery after photobleaching) analyses suggest that despite the shared role in pre-Start Cdc42 polarization, Rsr1 and Gic1/Gic2 may promote Cdc42 polarization via distinct mechanisms: the Rsr1 GTPase module links the spatial cue to Cdc42, and its function depends on local activation of Cdc42 by Bud3. In contrast, Gic1 and/or Gic2 interact with Cdc42-GTP and then stabilize Cdc42-GTP on the plasma membrane (PM), reducing lateral diffusion of Cdc42-GTP [61]. It is interesting to note that *rsr1*Δ cells display sporadic and relatively weaker pre-Start Cdc42 polarization compared to WT. Yet both WT and *rsr1*Δ cells display robust Cdc42 polarization in the second phase of G1 [46]. Pre-Start Cdc42 polarization by Gic1/Gic2 is likely essential in the absence of Rsr1 and thus in cells undergoing symmetry breaking. Interestingly, another study has also observed pre-Start Cdc42 polarization by locally recruiting Cdc24 by optogenetics in *rsr1*Δ cells. But recruitment of Bem1 before Start did not induce the positive feedback loop, suggesting that there are two distinct modes of Cdc42 polarization before and after Start [63]. Therefore, biphasic Cdc42 polarization is likely to occur in yeast cells regardless of their budding pattern.

While Rsr1 bound to GTP positively regulates polarity establishment, Rsr1 bound to GDP likely inhibits premature polarization through its interaction with the scaffold protein Bem1 [21]. Consistent with an earlier *in vitro* analysis [17], Rsr1 interacts with Bem1 preferentially in its GDP-bound state *in vivo*, and this interaction takes place during late M and early G1 phases [21]. Rsr1-GDP associates specifically with a part of the Bem1 Phox homology (PX) domain, which overlaps with a region previously shown to interact with Exo70, an exocyst component [64]. Furthermore, expression of a constitutively GDP-bound Rsr1 interferes with Bem1's association with Exo70, inhibiting Bem1-dependent Exo70 polarization, and also leads to delayed Cdc42 polarization and bud emergence. Consistent with these *in vivo* findings, mathematical modeling predicts that Bem1 is unable to promote Cdc42 polarization in early G1 in the presence of Rsr1-GDP. Thus, Rsr1-GDP likely interferes with the role of Bem1 in Cdc42 polarization and polarized secretion during the first phase of G1 in both haploid and diploid cells [21]. Importantly, this study suggests that Bem1-mediated positive feedback, which is critical for symmetry breaking, does not occur before Start because of its association with Rsr1-GDP, consistent with a previous finding that Bem1 functions in the polarity complex after Start [63] (see below). In contrast, another report argues that Bem1 and Cdc24 are active before Start in WT diploid cells [65]. The reason for this discrepancy is not clear, and further studies are required to clarify this issue.

### The second step leads to robust polarization to the incipient bud site

After Start, active Cdc28 (CDK1) complexed with a G1 cyclin (Cln1 or Cln2) phosphorylates Far1, a CDK1 inhibitor and a nuclear anchor of Cdc24, leading to subsequent degradation of Far1 and thus release of Cdc24 from the nucleus in haploid cells [42, 66]. Cdc24 is then directed to the selected site of polarized growth through association with Rsr1-GTP [16, 17, 67]. In the absence of spatial cues or a component of the Rsr1 GTPase module, yeast cells can still polarize Cdc42 at a single random site that is determined around the transition from the first to second temporal step in the G1 phase [46]. This implies that similar mechanisms may operate to reinforce polarity establishment after Start at a single chosen site during spatial-cue directed cell polarization as well as during symmetry breaking.

Previous studies on symmetry breaking have suggested that Bem1 mediates positive feedback for robust Cdc42 polarization [68, 69]. Bem1 was initially identified to play an essential role in symmetry breaking based on synthetic lethality of a *rsr1*Δ *bem1*Δ double mutant [68]. Bem1 was also found to be essential for cell polarization when actin is inhibited [69]. Expression of an artificial fusion protein of GEF Cdc24 – PAK Cla4 was able to rescue the lethality of the *rsr1*Δ *bem1*Δ mutant and drive polarization, establishing the role of the scaffold protein Bem1 in linking a Cdc42 GEF and a PAK for symmetry breaking [70]. A recent study employing an optogenetic strategy [63] provides a direct demonstration of the Bem1-mediated positive feedback loop. Remarkably, this study also suggests that the Bem1-mediated positive feedback loop requires CDK1-mediated phosphorylation: the Bem1 optogenetic construct could not induce Cdc42 cluster formation prior to the Start transition; and the Cdc24 optogenetic construct also could not recruit Bem1 prior to this transition [63]. Although it has not been directly tested whether this mechanism is also involved in spatial-cue directed cell polarization, these polarity factors are required for budding of WT cells. While a study argues that Bem1 promotes Cdc42 polarization prior to Start [65], an increasing number of evidence suggests that Bem1 functions after Start: the GEF activity of Cdc24 is enhanced by Bem1 [71, 72], which associates with Cdc24 after Start in cells lacking *RSR1* [63]. In addition, Bem1's role in promoting actin-independent localization of Exo70 is inhibited by Rsr1-GDP prior to Start [21] (see **Figure 4**).

Since Cdc42 promotes the organization of the actin cytoskeleton, which serves as tracks for the delivery of secretory vesicles, actin may also be involved in a positive feedback loop by promoting transport of Cdc42 on vesicles. However, the significance of actin-dependent feedback in polarity establishment is still under debate. A number of studies including mathematical modeling have suggested that the actin-dependent feedback loop acts in the generation of robust cell polarity [73, 74], which may function in parallel with the Bem1-mediated positive feedback [69]. Although Cdc42 trafficking on vesicles through endo- and exocytosis is slow, GDI (guanine nucleotide dissociation inhibitor)-mediated extraction of Cdc42-GDP can serve for fast recycling of Cdc42. It has been shown that GDI-mediated membrane-cytosol shuttling along with actin mediated delivery is necessary to establish a robust and stable Cdc42 polarity site [75, 76]. In contrast, a number of studies argue against the actin-dependent feedback for polarity establishment. These studies suggest that the slow delivery and low abundance of Cdc42 on polarized actin cables can lead to dilution of Cdc42 molecules at the polarity site rather than reinforcing polarization [77–81]. Interestingly, however, high-resolution imaging with complementary mathematical modeling indicates that the spatial coordination of opposing membrane trafficking activities via endocytosis and exocytosis allows robust polarity establishment, supporting the positive role of the actin cytoskeleton in polarity establishment [82]. While Cdc42-GTP auto-amplification can drive the clustering of exocytic activity to discrete sites, endocytic corralling ensures the selection of a unique, polarization cluster [82]. Additionally, actin depolymerization destabilizes the polarity cluster in both budding and fission yeasts [69, 83]. While expression of a GDI-insensitive *cdc42* allele is able to promote symmetry breaking, these budding yeast cells display a defect in proper bud-site selection and loss of singularity in budding [83]. Thus, the mechanisms promoting Cdc42 delivery to the PM may not be essential for initial establishment of cell polarity but may be important for robust Cdc42 polarization leading to bud emergence at a single site, which is particularly critical for the growth and division mode of budding yeast. While further investigation is necessary to fully understand these underlying mechanisms, actin-mediated positive feedback of Cdc42 polarization may be more important in the second temporal step of the G1 phase.

### Biological outputs of biphasic Cdc42 polarization

While we have a better understanding of the mechanisms underlying Cdc42 polarization, critical questions remain including why Cdc42 polarization occurs in two steps during G1. What are the biological outputs of stepwise Cdc42 polarization? In haploid **a** and α cells, Cdc42 polarization in the first phase of G1, which is triggered by Bud3, may be important for full assembly of the axial landmark and thus for establishing the polarity axis in the proper orientation. This idea has been supported by live-cell imaging and biochemical assays [37]. Each component of the axial landmark likely assembles sequentially at the division site during M and early G1 phase: Bud3 and Bud4 localize to the mother-bud neck first (i.e., future cell division site) through the interaction with septins [40, 84] and then recruit the other landmark proteins Axl1 and Axl2 [85]. Once this complex is fully assembled, it can interact with the Rsr1 GEF Bud5 [85, 86]. In contrast, in a *bud3* DH domain mutant with defective GEF activity, Bud4 fails to interact with Axl1, and Bud5 poorly associates with Bud4 and Axl1 [37]. Furthermore, Bud4 poorly associates with Axl1 in a temperature-sensitive *cdc42* mutant that is specifically defective in axial budding at a semi-permissive temperature, indicating that fully functional Cdc42 is necessary for axial landmark assembly [37]. Axial landmark assembly is likely critical for further recruitment and activation of Cdc42 via Rsr1 (and Bud3), leading to Cdc42 polarization at a single incipient bud site around Start. Active Rsr1 is then expected to guide Cdc24 and Cdc42 to the proper site for subsequent Cdc42 polarization in the second phase of G1 [16–20, 67]. Although Rsr1-GTP may also play a role in activation of Cdc24 by facilitating its release from autoinhibition [19], this role of Rsr1 is likely to be mediated by other regulators of Cdc24 [21, 71, 72] (see above).

Cdc42 also plays a key role in septin organization [87–89]. An earlier study by Bi and colleagues suggested that a new septin ring assembles stepwise – septins are first recruited as disorganized ‘clouds', which are then converted to a ring [87]. In a subsequent study, which combined live-cell imaging and computational modeling, they showed that septins recruited to the site of polarization by active Cdc42 subsequently inhibit Cdc42 in a negative feedback loop driven by Cdc42 GAPs. Polarized exocytosis then sculpts the septin patch into a ring, creating a hole that relieves inhibition of Cdc42 at the site of bud emergence [89]. Our recent study showed that this stepwise assembly of the septin ring occurs in correlation with biphasic Cdc42 polarization in the G1 phase [61]. When Rsr1 and both Gic proteins were depleted, cells failed to polarize Cdc42 during the first phase of G1 and as a consequence failed to recruit new septins. Remarkably, overexpression of Cdc42 allowed its own polarization as well as septin recruitment in the absence of Rsr1 and both Gic proteins [61]. Therefore, stepwise regulation of Cdc42 in relation to the two temporal steps of G1 is likely important to ensure the proper timing of events including the assembly and recognition of spatial landmarks, and execution of polarity establishment at a single site by organizing the actin and septin cytoskeleton (see **Figure 4**).

While an understanding of the mechanistic details requires further investigation, it is interesting to note that stepwise activation of Cdc42 and Rac GTPases has also been observed in other cell types, such as growth factor-stimulated endothelial cells and antigen-stimulated mast cells during spatial cue-directed cell polarization [90–92]. Therefore, biphasic activation of a GTPase may be a general mechanism underlying signal-responsive cell polarization.

## NEGATIVE POLARITY SIGNALING AND POTENTIAL IMPLICATIONS IN AGING

Cycling of the GTP- and GDP-bound states of Cdc42 is essential for proper cellular function in yeast and animals including humans. Thus, turning off Cdc42 activity is as important as its activation. Specific *cdc42* alleles that express dominant active mutant forms of Cdc42 cause dosage-dependent lethality in budding yeast [93]. Deletion of Cdc42 GAPs (*rga1*Δ *rga2*Δ *bem3*Δ), which leads to elevated levels of active Cdc42-GTP, also causes defects in septin organization and polarized growth in yeast, suggesting that GTP hydrolysis by Cdc42 is essential for its function [60, 88, 94].

Negative regulation of Cdc42 is also involved in selecting a new bud site. Both haploid and diploid cells select a new bud site that does not overlap with the previous bud site [95, 96]. Indeed, an elegant study showed that Rga1 plays a critical role in inactivating Cdc42 at the division site and thus blocks repolarization of Cdc42 within that site [44]. This distinct role of Rga1 in fine-tuning bud-site selection is not shared with other Cdc42 GAPs in haploid and diploid cells [44, 97]. Another negative regulator of Cdc42 signaling is Gps1 [98] (also known as Aim44, whose deficiency alters mitochondrial biogenesis and inheritance [99]). Gps1 establishes a novel polarity cue that sustains Rho1-dependent polarization but inhibits premature Cdc42-dependent activation of the PAK Cla4 at the site of cytokinesis [98]. Genetic analyses suggest that Gps1 may work in parallel with Rga1 to inhibit Cdc42 repolarization at the current division site [98]. These studies, however, did not answer why all old bud sites (other than the immediately preceding division site) are excluded from subsequent divisions.

**Figure 5:**
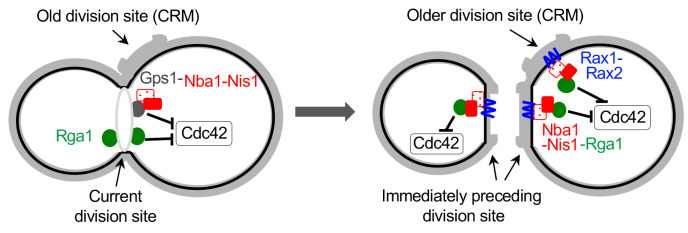
FIGURE 5: Model for inhibition of Cdc42 at the current and old cell division sites by negative polarity cues. During cytokinesis, Rga1 (green) and the Nba1-Nis1-Gps1 complex inhibit Cdc42 repolarization to the division site. After cytokinesis and septum formation, the Nba1-Nis1 complex (red) are inherited to the immediately preceding division site and remain at the older division sites (CRMs) via interaction with Rax1/Rax2 (blue lines). Rga1 also localizes transiently to CRMs via interaction with Nba1-Nis1 and inhibits Cdc42 repolarization at CRMs. The complex at the old division site is omitted in the cell shown on the left. Taken from Ref. [49].

All cell division sites are decorated with chitin-rich bud scars, which contain ‘cytokinesis remnants (CRM)' including Rax1 and Rax2, two interdependent transmembrane proteins. Rax1 and Rax2 localize to the distal pole (in daughter cells) as well as the division site after septation and stably remain at the site during multiple generations [100, 101]. Interestingly, while Gps1 does not localize to CRMs, it recruits Nba1 and Nis1, a negative polarity complex that antagonizes Cdc42 activation, at the current division site. Nba1 and Nis1 are subsequently inherited to CRMs and stably anchored via interaction with Rax1/Rax2 [34]. This negative polarity cue has been suggested to inhibit Cdc42 at CRMs by interfering with the interaction between Cdc24 and Rsr1 and thus locally preventing activation of Cdc24 [34]. However, this mechanism is debatable because neither Cdc24 nor Rsr1 localizes to CRMs. On the other hand, Rga1 localizes not only to the current cell division site but also to CRMs transiently [46, 49] via its direct interaction with Nba1 [49]. Genetic analyses also confirmed that Rga1 functions together with Nba1 and Nis1, rather than in parallel, in preventing re-budding within the current and all previous division sites [49]. Therefore, the inhibitory function of the negative polarity cues at CRMs is likely due to the GAP activity of Rga1, reinforcing the idea that Rga1 activity prevents re-use of any previous division site for yeast budding (**Figure 5**).

Remarkably, yeast cells lacking negative polarity cues display nuclear segregation defects and a decreased RLS [34]. These observations led to a proposal that cytokinesis remnants act as ‘cellular memory' for previous polarization events, and that negative polarity cues keep Cdc42 inactive at CRMs for proper asymmetric division [34]. The diameter of the bud neck region where nuclear segregation occurs is narrower in *rga1*Δ *nba1*Δ single or double mutant cells due to repeated re-budding at the same site. This abnormality has been attributed to the cause of impaired nuclear segregation and reduced lifespan in these mutants [34]. However, deletions of other Cdc42 GAPs (*rga2*Δ or *bem3*Δ) also display significantly reduced RLS than WT, even though their deletions do not have the same phenotypes as *rga1*Δ (PJK, unpublished). Thus, an enticing interpretation of these observations is that an increase in Cdc42 activity due to deletion of a Cdc42 GAP may contribute to cellular aging.

Cdc42 activity has been implicated in aging in other cell types. Notably, Cdc42 activity is substantially increased in hematopoietic stem cells as well as in other tissues from aged mice compared to those taken from young mice. This increased Cdc42 activity correlates with the depolarized phenotype and aging of hematopoietic stem cells [102]. Young hematopoietic stem cells divide mainly asymmetrically, whereas aged hematopoietic stem cells divide primarily symmetrically [103], as also observed in old yeast mother cells [33]. This mode of cell division is tightly linked to stem cell polarity and is regulated by the activity level of Cdc42 [103]. Large-scale data analyses and modeling have also found a correlation between *CDC42* upregulation in human white blood cells and aging as well as association of increased expression of *CDC42* with higher mortality [104]. Additionally, mice deficient for the p50RhoGAP protein, in which Cdc42 activity is increased in all tissues, present with premature aging-like phenotypes [105]. Interestingly, treatment of aged hematopoietic stem cells with a Cdc42 activity inhibitor (CASIN) reduced the level of Cdc42 activity to that of young cells and rejuvenated aged hematopoietic stem cells [102]. These studies raise a number of questions regarding the development of aging and reveal the intriguing possibility that negative regulation of Cdc42 is critical in aged cells as in young cells. While the connection between Cdc42 activity and aging is a tantalizing possibility, further investigation is necessary to reveal a concrete role of Cdc42 signaling in aging.

## CONCLUDING REMARKS

Cell polarity is a nearly universal feature that arises in almost all species ranging from bacteria to human and is fundamental to asymmetric cell division, growth, and development. Despite substantial progress in deciphering the mechanisms underlying Cdc42 polarization in budding yeast, significant gaps exist in our current knowledge of cell polarization. In particular, we are only beginning to understand how multiple processes leading to bud emergence, including actin and septin cytoskeleton organization and polarized secretion, are temporally coordinated. Another critical question is why budding yeast maintain such an elaborate program of polarized growth. Although a number of ideas have been suggested, it has not been directly addressed why this single-cell organism undergoes such complex oriented cell division as seen in developing embryos, which need a distinct body plan. To fully understand how yeast cells integrate multiple signals and why such a genetic program has evolved may benefit from both experimental and theoretical efforts. Further studies are required to understand the causative relationship of numerous changes associated with aging and the role of Cdc42 signaling. A deeper understanding of spatial and temporal regulation of Cdc42 activity in young and aged cells is undoubtably significant and may also lead to identification of key pharmacological targets for turning back the clock on aging.

## Abbreviations:

CDK: – cyclin-dependent kinase,
CRM: – cytokinesis remnants,
DH: – Dbl homology,
GAP: – GTPase activating protein,
GDI: – guanine nucleotide dissociation inhibitor,
GEF: – guanine-nucleotide exchange factor,
GTPase: – guanosine triphosphatase,
PM: – plasma membrane,
RLS: – replicative lifespan,
WT: – wild type.
